# Nutritional status of infants at six months of age following maternal influenza immunization: A randomized placebo-controlled trial in rural Nepal

**DOI:** 10.1016/j.vaccine.2017.09.095

**Published:** 2017-12-04

**Authors:** Joanne Katz, Janet A. Englund, Mark C. Steinhoff, Subarna K. Khatry, Laxman Shrestha, Jane Kuypers, Luke C. Mullany, Helen Y. Chu, Steven C. LeClerq, Naoko Kozuki, James M. Tielsch

**Affiliations:** a Johns Hopkins Bloomberg School of Public Health, Department of International Health, 615 N. Wolfe Street, Room W5009, Baltimore, MD 21205-2103, USA; b Seattle Children’s Hospital and Research Foundation, University of Washington, 4800 Sand Point Way N.E., R5441, Seattle, WA 98105, USA; c Global Health Center, Cincinnati Children’s Hospital Medical Center, 3333 Burnet Avenue, MLC2048, Cincinnati, OH 45229, USA; d Nepal Nutrition Intervention Project – Sarlahi, Kathmandu, Nepal; e Tribhuvan University, Department of Pediatrics and Child Health, Institute of Medicine, Kathmandu, Nepal; f School of Medicine, University of Washington, Molecular Virology Laboratory, Suite 320, 1616 Eastlake Ave. E., Seattle, WA 98102, USA; g University of Washington, Harborview Medical Center, 325 9th Ave, MS 359779, Seattle, WA 98104, USA; h Milken Institute School of Public Health, George Washington University, Department of Global Health, 950 New Hampshire Ave NW, Suite 400, Washington, DC, 20052, USA

**Keywords:** Maternal influenza vaccination, Early infancy, Birthweight, Small for gestational age, Stunting, Wasting, LBW, Low birth weight, WHO, World Health Organization, SGA, small for gestational age, AGA, appropriate for gestational age, RR, risk ratio, CI, confidence interval, BMI, body mass index

## Abstract

**Background:**

Maternal influenza vaccination has increased birth weight in two randomized trials in South Asia but the impact on infant growth is unknown.

**Methods:**

A randomized placebo-controlled trial of year round maternal influenza immunization was conducted in two annual cohorts in Sarlahi District, southern plains of Nepal, from April 2011 through April 2014. Infants born to women enrolled in the trial had weight, length, and head circumference measured at birth and 6 months of age. The study was powered for the 3 primary trial outcomes but not for stunting and wasting at 6 months of age.

**Results:**

3693 women received placebo or influenza vaccine between 17 and 34 weeks gestation, resulting in 3646 live births. About 72% of infants who survived had weight and length measurements between 150 and 210 days of age. Prevalence of stunting (<−2 Z scores length-for-age) was 14.8% in the placebo and 13.6% in the vaccine groups, respectively. Stunting < −3 Z scores was 3.2% versus 2.0% in placebo versus vaccine groups (RR: 0.64 (95% CI: 0.39, 1.04)). Wasting (< −2 Z scores weight for length) was 10.3% versus 11.0% for placebo versus vaccine groups. Severe wasting (< −3 Z scores weight for length) was 3.8% for placebo versus 2.6% for vaccine (RR: 0.69 (95% CI: 0.44, 1.07)). The impact of flu vaccine on wasting was greater in cohort 2 than in cohort 1, (RR: 0.66 (0.44, 0.99) for any wasting), and RR: 0.45 (0.19, 1.09) for severe wasting. This corresponded to a larger impact on birth weight and a better vaccine match with circulating viruses in cohort 2.

**Conclusions:**

Although maternal immunization reduced low birth weight by 15%, only wasting at 6 months in the 2nd cohort was statistically significantly difference. However, the study was underpowered to detect reductions of public health importance.

*Trial Registration:* Clinicaltrials.gov (NCT01034254).

## Highlights

• Maternal influenza vaccination reduced low birthweight by 15% in 2 annual cohorts randomized to vaccine or placebo.
• There was no difference in stunting among infants 6 months of age by treatment group.
• Wasting prevalence was lower in cohort 2 where vaccine matched circulating virus better.
• Severe wasting and stunting was lower in the vaccine group compared to placebo.

## 1 Introduction

Influenza may cause severe disease in all ages of the population worldwide, resulting in an estimated 19.2 million disability adjusted life years in 2010 [1]. Women who contract influenza during pregnancy have higher rates of morbidity and hospitalization than the general population, and infants born to these women are more likely to be preterm and of low birth weight (LBW, <2500 g) [2]. The impact of influenza disease during pregnancy and birth outcomes has generally been documented in high-income countries and most of the evidence for adverse effects on birth outcomes is among women with severe or pandemic influenza [3]. Influenza vaccination is recommended for pregnant women by the World Health Organization (WHO) to reduce maternal morbidity during pregnancy and potentially to protect infants from influenza early in life when morbidity is highest [4]. While this recommendation covers all countries, most low- and middle-income countries do not have policies or programs around maternal influenza vaccination. In observational studies, women vaccinated in pregnancy have been shown to have higher birth weight infants, reduced preterm births, LBW, and small-for-gestational age (SGA) but these studies are subject to significant confounding bias [5], [6]. Recent large randomized controlled trials in Bangladesh, Mali, South Africa and Nepal have examined the impact of maternal influenza vaccination on maternal and infant influenza [7], [8], [9], [10], [11], [12]. The Bangladesh and Nepal trials both showed impacts on birth weight, whereas those in Mali and South Africa did not. The trial in Nepal did not find an impact on preterm births or SGA overall but did find a greater birth weight effect during high circulation of influenza and when there was a match between vaccine strains and those in circulation [13]. Given the impact of LBW and gestational age on infant growth, we sought to examine whether the positive effect of maternal influenza vaccination on birth weight had longer term advantages for infant growth at 6 months of age in the trial we undertook in the southern plains of rural Sarlahi district, Nepal.

## 2 Methods

A randomized placebo controlled trial of year round maternal influenza vaccination versus saline placebo was conducted in Nepal from April 2011 with follow up through April 2014, when enrolled women and their infants had been followed through 6 months postpartum. The trial is registered at clinicaltrials.gov (NCT01034254). Methods are described in detail elsewhere [11], [12]. Briefly, the trial was conducted in 9 Village Development Committees of Sarlahi district. Data collectors identified households with women 15–40 years of age and visited them every 5 weeks to ask about menses and provide pregnancy tests for women who had not menstruated since the last visit. Date of last menstrual period was recorded at each 5 weekly visit. All pregnant women in this geographic area were approached to participate in the trial. They were consented and individually randomized in blocks of 8, stratified by gestational age at vaccination (17–25 and 26–34 weeks), to influenza vaccine or a saline placebo in two cohorts. The first cohort was enrolled and vaccinated from April 25, 2011 through April 24, 2012. The second cohort was enrolled between April 25, 2012 and April 24, 2013, but vaccinations in cohort 2 were assigned to be administered randomly between 17 and 34 weeks gestation. Hence, vaccinations occurred between April 25, 2012 and September 9, 2013. Vaccine used was the inactivated trivalent influenza Vaxigrip® vaccine (Sanofi Pasteur, Ltd.). Follow-up of all vaccinated mother-infant dyads through 6 months post-partum ended April 2014. The intervention was masked to everyone except those administering the vaccinations. Following vaccination, women were visited in their homes weekly through 6 months post-partum. At these visits, women were asked to recall their own respiratory symptoms in the prior week, and following delivery, of their infant(s). Women with fever and other respiratory symptoms had a mid-nasal swab collected. Infants with fever or any other respiratory symptoms were also swabbed. Specimens were tested for influenza, pertussis and a panel of other respiratory viruses using RT-PCR [14]. Primary outcomes of the trial were influenza-like illness in pregnancy, low birth weight, and laboratory confirmed influenza in infants in the first 6 months of life. The impact of vaccination on infant nutritional status at 6 months of age was a secondary outcome. However, the trial was not powered to address the secondary aims. Gestational age was estimated using prospectively collected dates of last menstrual period and date of birth.

The sample size of 1850 women in each of the two trial cohorts was based on detecting a 50% reduction in laboratory confirmed influenza in infants, a 33% reduction in influenza like illness in women and a 25% reduction in LBW with an overall type I error of 5% (0.017 for each of the 3 outcomes) and power of 90% for each trial cohort separately. The study was powered for the 3 primary trial outcomes but not for differences of public health importance in 6 month nutritional status.

As soon after birth as possible, data collectors visited the homes of newborns to record the outcome of the pregnancy, weigh the newborn and measure length and head circumference. The same measurements were taken at 6 months post-partum prior to discharge from the trial. Weights at both times were measured with a digital infant scale, either the Seca model 727 (measuring to the nearest 2 g) or the Tanita model BD-585 (measuring to the nearest 10 g). Scales were standardized daily with standard weights before use. Length was measured using a Shorr length board to the nearest 0.1 cm. Three measures were taken and the median recorded for analysis. Head circumference was measured using a Kendall mid upper arm circumference tape to the nearest 0.1 cm. The tape was firmly placed around the frontal bones just superior to the supra-orbital ridges, passed around the head at the same level on each side, and laid over the maximum occipital prominence at the back of the head. The median of three measurements was recorded for analysis. Measurements taken within 72 h of birth were used to estimate birth weight, low birth weight (<2500 g), birth length, head circumference at birth, and SGA (infants below the 10th percentile of the Intergrowth International fetal growth standard) [15]. For 6-month follow up, measures were used if taken between 150 and 210 days after birth.

Characteristics of infants whose mothers received placebo and flu vaccine were compared among infants who had anthropometric measurements at 6 months of age. The same characteristics were also compared between all live births who did and did not have 6 month anthropometry. Among those with 6 month anthropometry, the impact of the vaccine was estimated by calculating the mean difference and 95% confidence intervals in weight, height and head circumference between treatment groups. These measures were not adjusted for infant age because the mean age of measurement in each group was identical (175 days). The WHO growth standards were used to calculate length-for-age and weight-for-length z-scores (ref WHO standards) [16]. These growth standards are age and sex specific. Any weights and lengths for which the Z-scores were <−7 or ≥7 or missing (2 infants were missing sex) were excluded from the analysis. The impact of the vaccine on stunting (<−2 z-scores length for age), severe stunting (<−3 Z-scores), wasting (<−2 Z-scores weight for length) and severe wasting (<−3 Z-scores) was compared by calculating risk ratios and 95% confidence intervals from log-binomial regression models. These treatment effects were also stratified by LBW, SGA, preterm, infant influenza, and sex of the infant. We also examined the impact of LBW and influenza infection in the first six months of life on growth of infants to assess the extent to which these two factors associated with vaccine efficacy might contribute to 6-month growth. Analyses were conducted for each cohort separately and for the two cohorts combined, as was done in the primary outcomes paper [12].

To examine the impact of differences between infants who did and did not have anthropometry at 6 months of age, we imputed missing lengths and weights as a sensitivity analysis only. The following predictor variables were used: age of the child (in days) at the six-month follow-up visit, birthweight, gestational age at birth, sex, death within first six months of life, maternal height (<150 cm vs. ≥150 cm), primiparity, vaccination status (mother received influenza vaccine vs. placebo from study), and gestation in pregnancy at which the vaccine was received. The multiple imputation command “mi” in Stata was used to create 20 imputations which were pooled according to the approach of Rubin [17]. The Z-scores for weight-for-length and length-for-age were also imputed and then dichotomized into binary variables of <−2 vs. ≥−2 Z-scores and <−3 vs. ≥−3 Z-scores respectively.

Informed consent was obtained from all participants. This trial protocol was approved by the Institutional Review Boards of the Cincinnati Children’s Medical Center, Johns Hopkins Bloomberg School of Public Health (JHBSPH), Institute of Medicine at Tribhuvan University, Kathmandu, Nepal, and the Nepal Health Research Council. IRBs at Seattle Children’s Hospital, the University of Washington, and George Washington University granted oversight to the IRB at JHBSPH. A Data Safety and Monitoring Board reviewed adverse events throughout the trial but no interim outcome analyses were conducted.

## 3 Results

**Fig. 1:**
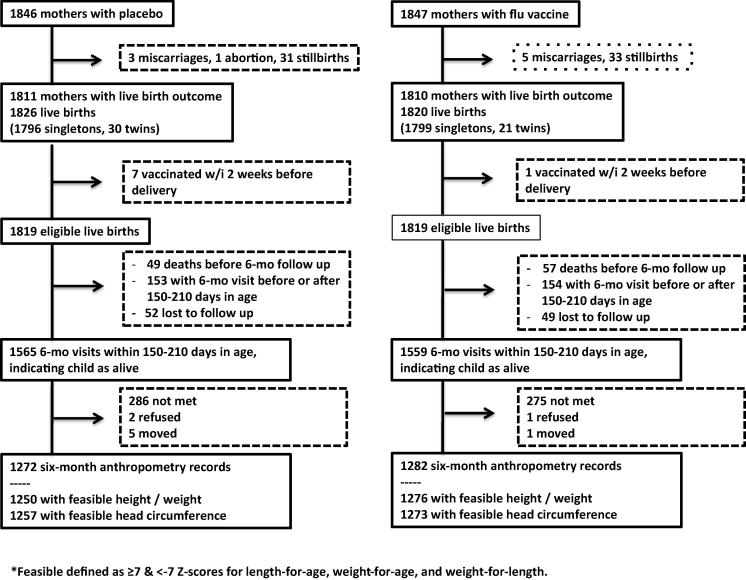
Flow diagram of participation and follow up of infant growth in the randomized controlled trial of maternal influenza vaccination in rural Nepal.

A total of 3693 women were randomized to placebo (1846) or influenza vaccine (1847), resulting in 1826 (placebo) and 1820 (influenza) livebirths (Fig. 1). 8 infants were removed from the analysis because their mothers received the vaccine within two weeks of delivery and thus were not expected to have responded fully to the vaccine. Among live born infants who survived through 180 days of age, feasible lengths and weights were measured within 150–<120 days of age for 70.7% of placebo and 72.7% of flu vaccine infants.

Characteristics of infants with anthropometry at six months were comparable between those whose mothers received placebo and those who received flu vaccine (Table 1). Among those live births without 6-month anthropometry, infants of women who received flu vaccine had birth weights 87 g heavier than those who received placebo (Supplemental Table 1). In addition, the percent LBW and SGA were lower for infants without anthropometry whose mothers received flu vaccine compared with placebo infants. Other characteristics were comparable between treatment groups for those who did not have 6-month anthropometry. Placebo infants without anthropometry at 6-months (including 49 infants who died prior to the 6 month follow up visit) had lower mean birth weights (69 g, p = 0.013), higher% LBW (p = 0.005) and preterm (p = 0.016), lower % vaccinated earlier in pregnancy (17–<25 weeks, p < 0.001), and nulliparous (p = 0.025) than placebo infants measured at 6 months (Table 1 and Supplemental Table 1). Among infants whose mothers received flu vaccine, the infants with and without anthropometry were statistically significantly comparable although comparisons of some of these characteristics may have been underpowered given the sample size. The mean age at which infants had anthropometry taken was 175 days in both groups.

**Table 1:** Baseline characteristics of infants with anthropometry at 6 months of age whose mothers received placebo or flu vaccine.

	Placebo (n = 1250)		Vaccine (n = 1276)	
	N	% or mean (SD)	N	% or mean (SD)
*Infant characteristics*				
Sex	1250		1276	
Male		50.7		54.2
Female		49.3		45.8
Birthweight in grams (taken within 72 h)	997	2780 (450)	1032	2806 (450)
Gestational age in weeks (within feasibility range of 23–<50 weeks)	1247	39.4 (2.4)	1274	39.5 (2.4)
LBW	997	24.9	1032	22.9
SGA (Intergrowth)^19^	955	36.7	982	36.1
Preterm	1247	12.3	1274	11.8
Age when six-month anthropometry taken, in days	1250	175 (10)	1276	175 (10)

*Maternal characteristics at time of vaccination*				
Age (years)	1250	23.1 (4.6)	1276	23.3 (4.7)
Gestational age at vaccination (weeks)	1249	23.2 (5.1)	1276	23.2 (5.3)
<26		65.4		64.6
≥26		34.6		35.4
Systolic BP, at enrollment (mm Hg)	1244	100.2 (9.7)	1272	100.3 (9.5)
Diastolic BP, at enrollment (mm Hg)	1244	66.4 (8.5)	1272	66.5 (9.1)
Height, at enrollment (cms)	1246	151.8 (5.5)	1272	151.9 (5.5)
Weight, at enrollment (kgs)	1247	48.1 (7.2)	1273	48.7 (7.8)
BMI, at enrollment	1246	20.8 (2.7)	1272	21.1 (2.9)
<18.5		19.0		17.5
18.5–<25		73.0		73.7
25–<30		7.5		7.6
30+		0.4		1.3
No education	1181	59.6	1211	58.8
Nulliparous	1247	41.1	1273	40.1

*Includes children who contributed feasible height and weight data, had to be age 150–<210 days when measurement taken.

There were no statistically significant differences in mean weight or length at six months of age between vaccine and placebo groups, and no differences between groups when stratified by LBW, preterm, SGA or trial cohort (Table 2). However, the 95% confidence limits for the difference in mean weight at 6 months of age were consistent with up to a 65 g lower weight among vaccinated infants than those receiving placebo and up to a 90 g higher mean weight among vaccinated infants.

**Table 2:** Six-month weights and lengths of live born infants by treatment group and vaccine impact on weights and lengths by birth outcomes and cohorts.

		Weight (grams)	Length (cms)
	N	Mean (SD)	Mean (SD)
Placebo	1250	6791 (1015)	64.3 (2.5)
Vaccine	1276	6803 (9 7 1)	64.4 (2.4)

		Vaccine-PlaceboMean Diff (95% CI)	Vaccine-PlaceboMean Diff (95% CI)

Overall effect		12.5 (−64.9, 90.0)	0.06 (−0.13, 0.25)

LBW*	484	12.4 (−142.8, 167.6)	0.38 (−0.05, 0.80)
Non-LBW	1545	−19.6 (−112.9, 73.7)	−0.10 (−0.32, 0.12)

Preterm	303	63.0 (−162.4, 288.5)	0.33 (−0.24, 0.91)
Term	2218	−0.6 (−82.7, 81.5)	0.00 (−0.20, 0.21)

SGA*	704	−14.2 (−151.7, 123.2)	0.02 (−0.34, 0.38)
AGA	1233	−3.4 (−108.6, 101.8)	0.07 (−0.19, 0.32)

Cohort 1	1528	−5.5 (−105.1, 94.1)	0.16 (−0.09, 0.41)
Cohort 2	998	41.6 (−81.9, 165.0)	−0.11 (−0.41, 0.19)

* Overall effect includes all infants with 6 month anthropometry but LBW and SGA includes only those with birth weight within 72 h.

The mean 6 month weights and lengths were plotted by calendar time overlaid onto the intensity of circulation of influenza virus by strain (Fig. 2a, Fig. 2b). The peak season of circulation was approximately June/July/August each year although a low level of circulation continued during many other months of the year. In terms of a match between vaccine protection and circulating virus strains, only two vaccines were used during the trial. The first contained protection against Perth A/H3N2, California A/H1N1 and B/Brisbane (Victoria) strains and was used from April 25, 2011 through October 20, 2012. The second contained B/Brisbane Victoria, A/H3N2, California A/H1N1, and B/Wisconsin (Yamagata), and was used from October 21, 2012 through September 9, 2013. As can be seen on the figures, B/Wisconsin (Yamagata) circulated from November 2011 through January 2012 and August through October 2012, which was prior to the switch from the first to the second vaccine. During that time, the vaccine strain (B/Victoria) did not antigenically match the circulating strain (B/Yamagata) and good protection against influenza B was not seen in our study during those times. Children born to mothers who received flu vaccine at the start of the influenza season (June/July with 6 month old measurements in December/January) did not appear to have higher weights and lengths than those who mothers received placebo. Children growing when B/Wisconsin (Yamagata) was circulating and the vaccine did not contain this antigen had similar attained mean weights and lengths as those growing during periods when the vaccine contained antigens matching the circulating virus strains. Mean 6 month weight was 30.3 g (95% CI: −60.3, 120.8) greater in vaccinated versus placebo infants during the period where the vaccine did not protect against B Yamagata and 29.0 g (95% CI: −26.9, 84.9) greater among vaccinated versus placebo infants in the time periods where the vaccine did protect against circulating viruses. Similarly, 6 month length was 0.03 (95% CI: −0.15, 0.20) greater during the period when the vaccine did not protect against B Yamagata and 0.03 cm (95% CI: −0.13, 0.20) less in the vaccine compared to placebo during the period when the vaccine was protective against circulating strains.

**Fig. 2a:**
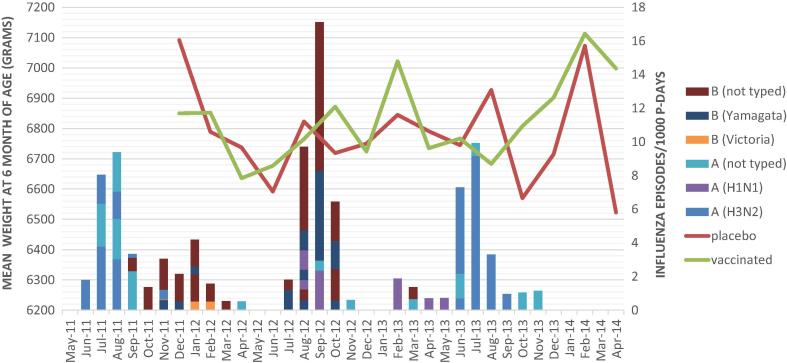
Mean weight at 6 months of age in grams (left Y axis) for infants born to mothers who received vaccine or placebo in pregnancy by calendar time (X axis) overlaid on incidence of circulating influenza strains (right Y axis).

**Fig. 2b:**
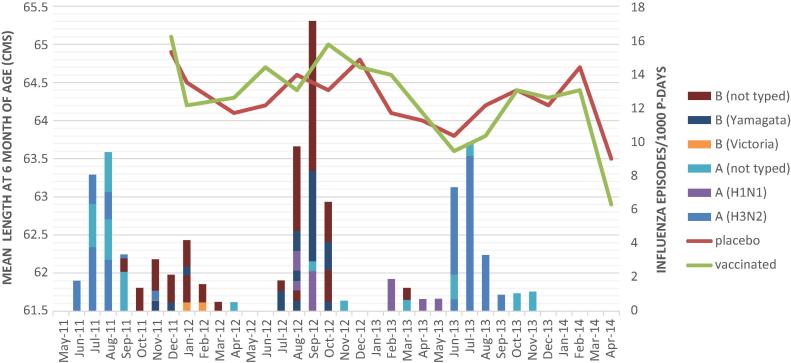
Mean length at 6 months of age in centimeters (left Y axis) for infants born to mothers who received vaccine or placebo in pregnancy by calendar time (X axis) overlaid on incidence of circulating influenza strains (right Y axis).

There were no statistically significant differences in the proportion stunted or wasted at 6 months between the placebo and vaccine groups (Table 3). However, the 95% confidence intervals were consistent with up to a relative reduction in wasting of 15% and in stunting of 24%. Maternal influenza vaccine was protective against severe wasting (RR: 0.69 (0.44, 1.07)) and severe stunting (RR: 0.64 (0.39, 1.04)) although not statistically so. While LBW and preterm infants whose mothers received flu vaccine had lower severe stunting than those who received placebo, these differences were not statistically significant. Among appropriate for gestational age (AGA) infants, the flu vaccine significantly reduced stunting (RR: 0.71 (0.51, 0.99)). The impact of flu vaccine on wasting was greater in cohort 2 than in cohort 1 (cohort 2 also had the larger birth weight effect), with RR: 0.66 (0.44, 0.99) for any wasting, and RR: 0.45 (0.19, 1.09) for severe wasting. There were no differences in vaccine effect on anthropometric outcomes by sex of the infant, or by whether the infant had laboratory confirmed influenza in the first six months of life (data not shown). Mean head circumference of placebo and vaccine infants were not different at birth or at six months of age (32.8 cm at birth and 41.1 cm at six months in the placebo group and 32.9 cm at birth and 41.2 cm at six months in the vaccinated group).

**Table 3:** Six-month stunting and wasting among live born infants by treatment group and vaccine impact on wasting and stunting by birth outcomes and cohorts.

		Wasting	Stunting
Z-score < −2	N	%	%
Placebo	1250	10.3	14.8
Vaccine	1276	11.0	13.6
		RR (95% CI)	RR (95% CI)
		1.06 (0.85, 1.33)	0.92 (0.76, 1.12)

LBW	484	1.24 (0.84, 1.83)	0.91 (0.68, 1.21)
Non-LBW	1545	1.04 (0.76, 1.44)	0.92 (0.67, 1.24)

Preterm	303	1.38 (0.77, 2.48)	0.92 (0.62, 1.36)
Term	2218	1.04 (0.81, 1.32)	0.95 (0.76, 1.18)

SGA	704	1.18 (0.84, 1.64)	1.03 (0.77, 1.38)
AGA	1233	1.09 (0.75, 1.58)	**0.71 (0.51, 0.99)**

Cohort 1	1528	**1.34 (1.01, 1.78)**	0.82 (0.62, 1.08)
Cohort 2	998	**0.66 (0.44, 0.99)**	1.06 (0.81, 1.38)

Z-score < −3	N	%	%

Placebo	1250	3.8	3.2
Vaccine	1276	2.6	2.0
		RR (95% CI)	RR (95% CI)
		0.69 (0.44, 1.07)	0.64 (0.39, 1.04)

LBW	484	1.24 (0.84, 1.83)	0.67 (0.35, 1.28)
Non-LBW	1545	1.04 (0.76, 1.44)	0.71 (0.25, 2.02)

Preterm	303	1.38 (0.77, 2.48)	0.61 (0.23, 1.64)
Term	2218	1.04 (0.81, 1.32)	0.67 (0.38, 1.18)

SGA	704	0.90 (0.51, 1.60)	0.66 (0.32, 1.35)
AGA	1233	0.69 (0.031, 1.54)	0.53 (0.20, 1.41)

Cohort 1	1528	0.79 (0.48, 1.32)	0.62 (0.32, 1.17)
Cohort 2	998	0.45 (0.19, 1.09)	0.67 (0.32, 1.41)

In order to examine mechanisms by which poorer growth in the first six months of life might occur, we compared the attained mean 6 month weight and length of LBW infants to those of normal birth weight, adjusted for maternal height, parity, infant sex, age at first marriage and treatment group. LBW infants attained an adjusted 1.59 (95% CI: −1.81, −1.38) cm and 698 g (95% CI: −786, −609) less than normal birth weight infants (Table 4). LBW infants also had significantly higher rates of stunting and wasting at six months compared to normal birth weight infants (Table 5). Similarly, infants with laboratory confirmed influenza gained less weight and length but these differences were much smaller than for LBW and not statistically significant (Table 4, Table 5).

**Table 4:** Impact of low birth weight and laboratory-confirmed influenza incidence on attained weight and length of infants in the first 6 months of life, adjusted.

		Weight (g)	Length (cm)
	N	Mean (SD)	Mean (SD)
LBW	481	6188 (869)	62.9 (2.4)
Non-LBW (ref)	1537	6935 (935)	64.7 (2.2)
		Adjusted Mean Diff (95% CI)	Adjusted Mean Diff (95% CI)
	2018	**−697.9 (−786.3, −609.4)**	**−1.59 (−1.81, −1.38)**
Influenza	125	6681 (943)	64.0 (2.2)
No Influenza (ref)	2389	6802 (996)	64.4 (2.5)
		Adjusted Mean Diff (95% CI)	Adjusted Mean Diff (95% CI)
	2514	−92.0 (−257.7, 73.8)	−0.27 (−0.67, 0.14)

*Adjusted for maternal height, parity, infant sex, vaccination status, age at first marriage

**Table 5:** Impact of low birth weight and laboratory-confirmed influenza incidence on stunting and wasting of infants in the first 6 months of life, adjusted.

		Wasting	Stunting
	N	%	%
z-score < −2			
LBW	481	17.5	28.3
Non-LBW (ref)	1537	8.7	9.6
		Adjusted RR (95% CI)*	Adjusted RR (95% CI)*
	2018	**2.06 (1.60, 2.65)**	**2.88 (2.34, 3.54)**
Influenza	125	10.4	20.0
No Influenza (ref)	2389	10.6	14.0
		Adjusted RR (95% CI)*	Adjusted RR (95% CI)*
	2514	0.91 (0.54, 1.55)	1.30 (0.92, 1.86)

z-score < −3			
LBW	481	5.8	7.5
Non-LBW (ref)	1537	2.7	0.9
		Adjusted RR (95% CI)*	Adjusted RR (95% CI)*
	2018	**2.15 (1.33, 3.46)**	**8.05 (4.47, 14.50)**

Influenza	125	4.0	4.8
No Influenza (ref)	2389	3.1	2.5
		Adjusted RR (95% CI)*	Adjusted RR (95% CI)*
	2514	1.12 (0.46, 2.74)	1.67 (0.76, 3.66)

* Adjusted for maternal height, parity, infant sex, vaccination status, age at first marriage.

Imputation of missing weights and lengths at 6 months increased the weights and lengths available from n = 2526 to n = 3197. When the imputed weights, lengths, weight-for-length Z-scores, and length-for-age Z-scores were used in the regression analyses, there were no changes in the inferences from the analyses conducted without the imputed values (Supplemental Table 2).

## 4 Discussion

We have previously shown that influenza vaccine reduced influenza-like illness in pregnancy, laboratory-confirmed influenza in infants during the first six months of life in southern Nepal, LBW by 15%, and modestly increased birth weight [12]. In this analysis of infant anthropometry at six months of age, there was no evidence of a statistically significant reduction in mild to moderate stunting or wasting at six months of age. Although the point estimates of the differences between the vaccine and placebo groups were not of public health importance, the confidence intervals for these effects were consistent with impacts of importance that may have been detected with a larger sample size. The vaccine impact on severe stunting and wasting was larger but these were meaningful public health differences which we did not have the power to detect given the sample size and the relatively low prevalence of severe stunting and wasting. The vaccine did have a statistically significant 34% reduction in wasting and a 55% reduction in severe wasting in the 2nd annual cohort. This corresponds with the larger impact on birth weight in the second cohort of 62 grams as well as a better vaccine match during part of this time period.

These results are likely generalizable to rural populations of South Asia among women of low stature and body mass index (BMI), which may have limited the ability to observe a growth effect on infants. There was also little evidence that maternal influenza vaccine benefited any subgroups of infants over others. In particular, infants with adverse birth outcomes such as being born LBW or preterm were not seen to benefit more or less than those without these conditions, although the study was not powered to detect such effect modifications. Although LBW infants had lower attained weight and length than normal birth weight infants at 6 months of age, the impact of the vaccine on 6 month anthropometry did not differ by whether the infant was born LBW or normal birth weight.

Among infants with 6-month anthropometric measurements, treatment groups were comparable with regard to birth outcomes and maternal characteristics that might predict nutritional status at six months of age. Overall, 28% of trial infants were missing anthropometry at the six-month visit. Infants in the vaccine group without 6-month data were comparable to those with anthropometry at six months of age. Infants in the placebo group who did not have six-month visits were found to have somewhat poorer birth outcomes than those who had six-month follow-up. Using multiple imputation of missing 6 month weights and lengths, the poorer birth outcomes and maternal characteristics did not appear to change the inferences about treatment effect on nutritional status at 6 months. However, it is possible that the multiple imputation did not include some relevant variables that may have identified biases in the vaccine impact on 6 month anthropometry if included.

Maternal influenza or influenza-like illness during pregnancy may increase inflammation and reduce appetite, leading to lower weight gain in pregnancy, poorer intrauterine growth, LBW, and possibly preterm delivery [2], [18]. In our trial, the impact of vaccination on low birth weight was a 15% reduction in both cohorts over the three years of the trial, but with a higher difference in mean birth weight of 62 grams in the second cohort, possibly associated with better match between vaccine and circulating virus types [16]. There was no impact of the vaccine on preterm or gestational age. The greatest impact of vaccination on birth weight occurred among infants who were both SGA and preterm [13]. Reductions in more severe wasting and stunting at 6 months were consistent with the vaccine’s impact on birth weight and LBW, especially in the 2^nd^ annual cohort, but the sample size and prevalence of severe wasting and stunting limited our ability to detect these differences statistically. Mechanisms by which maternal vaccination might improve infant growth during the first 6 months of life include improved birth weight leading to improved postnatal growth and/or fewer infections in general, or a direct effect of fewer influenza infections early in life. However, influenza is relatively uncommon in young infants in this area of Nepal (5.8% attack rate among those in the placebo group) and the vaccine averted only 56 cases of influenza per 1000 child-years. Hence, at a population-level, such an impact is unlikely to translate into a growth effect, although our data suggest it may at the more extreme end of the malnutrition spectrum. Other mechanisms could be postulated such as the potential impact of fever and increased cytokine release resulting in inflammatory cascades.

Although this study did not find a statistically significant impact of the vaccine on infant stunting and wasting at 6 months of age, the limits of confidence intervals were consistent with impacts of public health importance, indicating inadequate sample size to refute the hypothesis of no effect. In addition, sizeable point estimates of the vaccine effect were seen for severe wasting and stunting, but the study was underpowered to detect these differences. Another limitation is that although 6 month anthropometry was obtained in 70% of infants overall, 28% of infants for whom anthropometry was not obtained at six months of age had poorer birth outcomes in the placebo group relative to those with six month follow up. This could have led to some bias in our vaccine effect estimates. Multiple imputation of the missing 6 month anthropometry did not indicate such a bias but this analysis is limited by the availability of predictive variables for such imputation. Strengths of this study include a large population-based sample of infants enrolled in a randomized, placebo controlled trial, weight measurements taken with accurate scales, and the median of three length and head circumference measurements used in analysis.

The data from this randomized placebo controlled trial suggest that while maternal influenza vaccination did improve birth weight and reduce infant influenza infections, these effects were not large enough to translate into an impact on mild to moderate malnutrition of the population through six months of life, although there was some evidence it reduced severe malnutrition, especially during time periods where the vaccine provided protection against circulating strains. Given a lack of randomized trial data of the impact of maternal vaccination on infant growth and the sample size limitations of this trial, further studies designed to address malnutrition in young infants as a primary outcome are needed.

## Authors’ Contributions

Mark C. Steinhoff, James M. Tielsch, Joanne Katz, Janet A. Englund, and Jane Kuypers contributed to the design of the study.

Mark C. Steinhoff conceived of the study and secured funding for the project.

Subarna K. Khatry, Steven C. LeClerq, Laxman Shrestha, James M. Tielsch, Luke C. Mullany and Joanne Katz supervised the conduct of the study in the field.

Joanne Katz drafted the manuscript, served as the study statistician, and Joanne Katz and Luke C. Mullany designed/implemented the data analysis.

Helen Y. Chu assisted in study implementation and cord blood collection.

Naoko Kozuki provided data analysis support.

## Conflicts of interest

Joanne Katz, James Tielsch, Subarna Khatry, Mark Steinhoff, Steven LeClerq, Laxman Shrestha, Naoko Kozuki, Luke Mullany, Helen Chu and Jane Kuypers have no potential conflicts of interest. Janet Englund has been a consultant for Pfizer, a member of a Data Safety Monitoring Board for GlaxoSmithKline (GSK) influenza antiviral studies, and her institution has received research support for clinical studies from GSK, Gilead, Chimerix, and Roche.

All authors read and approved the final manuscript.

## References

[1] Murray C.J., Vos T., Lozano R. (2012). Disability-adjusted life years (DALYs) for 291 diseases and injuries in 21 regions, 1990–2010: a systematic analysis for the Global Burden of Disease Study 2010. Lancet.

[2] Rasmussen S.A., Jamieson D.J., Uyeki T.M. (2012 Sep). Effects of influenza on pregnant women and infants. Am J Obstet Gynecol..

[3] Fell D.B., Savitz D.A., Kramer M.S. (2017 Jan). Maternal influenza and birth outcomes: systematic review of comparative studies. BJOG.

[4] Vaccines against influenza (2012). WHO position paper – Weekly Epidemiological Record.

[5] Vazquez-Benitez G., Kharbanda E.O., Naleway A.L. (2016 Aug 1). Risk of preterm or small-for-gestational-age birth after influenza vaccination during pregnancy: caveats when conducting retrospective observational studies. Am J Epidemiol.

[6] Savitz D.A., Fell D.B., Ortiz J.R., Bhat N. (2015 Nov 25). Does influenza vaccination improve pregnancy outcome? Methodological issues and research needs. Vaccine.

[7] Omer S.B., Richards J.L., Madhi S.A. (2015). Three randomized trials of maternal influenza immunization in Mali, Nepal, and South Africa: Methods and expectations. Vaccine.

[8] Steinhoff M.C., Omer S.B., Roy E. (2012). Neonatal outcomes after influenza immunization during pregnancy: a randomized controlled trial. CMAJ.

[9] Tapia M.D., Sow S.O., Tamboura B. (2016). Maternal immunisation with trivalent inactivated influenza vaccine for prevention of influenza in infants in Mali: a prospective, active-controlled, observer-blind, randomised phase 4 trial. Lancet Infect Dis.

[10] Madhi S.A., Cutland C.L., Kuwanda L. (2014 Sep 4). Influenza vaccination of pregnant women and protection of their infants. N Engl J Med.

[11] Tielsch J.M., Steinhoff M., Katz J. (2015). Designs of two randomized, community-based trials to assess the impact of influenza immunization during pregnancy on respiratory illness among pregnant women and their infants and reproductive outcomes in rural Nepal. BMC Pregnancy Childbirth.

[12] Steinhoff MC, Katz J, Englund JA, et al. Year-round influenza immunization in pregnancy: a randomized placebo-controlled trial in Nepal. Lancet Inf Dis 2017;May 15. doi:10.1016/S1473-3099(17)30252-9 [EPub ahead of print]. 10.1016/S1473-3099(17)30252-9 PMC5573632 28522338

[13] Kozuki N., Katz J., Englund J.A. (2017). Impact of maternal vaccination timing and influenza virus circulation on birth outcomes in rural Nepal. Int J Gynaecol Obstet.

[14] Kuypers J., Wright N., Ferrenberg J. (2006). Comparison of real-time PCR assays with fluorescent-antibody assays for diagnosis of respiratory virus infections in children. J Clin Microbiol.

[15] Villar J., Cheikh Ismail L., Victora C.G. (2014 Sep 6). International standards for newborn weight, length, and head circumference by gestational age and sex: the Newborn Cross-Sectional Study of the INTERGROWTH-21st Project. Lancet.

[16] World Health Organization. WHO child growth standards: height-for-age, weight-for-age, weight-for-length, weight-for-height and body mass index-for-age: methods and development Geneva: WHO; 2006. Available: http://www.who.int/childgrowth/standards/technical_report.

[17] Rubin D.B. (1996). Multiple imputation after 18+ years. J Am Stat Assoc.

[18] Christian P. (2014). Fetal growth restriction and preterm as determinants of child growth in the first two years and potential interventions. Nestle Nutr Inst Workshop Ser.

