# Supplementary material for: Nutritional status of infants at six months of age following maternal influenza immunization: A randomized placebo-controlled trial in rural Nepal
Source: Vaccine. 2017 Dec 4;35(48Part B):6743–50. doi: 10.1016/j.vaccine.2017.09.095 (PMC5714610; doi:10.1016/j.vaccine.2017.09.095)
Supplement: Supplementary data 1 [file mmc1.docx]

Supplemental Table 1: Baseline characteristics of live born infants without anthropometry at 6 months of age whose mothers received placebo or flu vaccine

|  | placebo (N=568) | | | vaccine (N=535) | | |
| --- | --- | --- | --- | --- | --- | --- |
|  | N | % or mean(SD) | p* | N | % or mean(SD) | p* |
| **Infant characteristics** | | | | | | |
| Sex | 566 |  | 0.489 | 535 |  | 0.763 |
| Male |  | 52.5 |  |  | 53.5 |  |
| Female |  | 47.5 |  |  | 46.5 |  |
| Birthweight (taken within 72 hours) | 364 | 2711 (469) | 0.0125 | 346 | 2798 (452) | 0.7914 |
| Gestational age (within feasibility range of 23-<50 weeks) | 565 | 39.2 (2.8) | 0.1858 | 535 | 39.3 (2.7) | 0.3254 |
| LBW | 364 | 32.4 | 0.005 | 346 | 22.8 | 0.989 |
| SGA (Intergrowth) | 345 | 41.2 | 0.139 | 323 | 32.2 | 0.208 |
| Preterm | 565 | 16.5 | 0.016 | 535 | 14 | 0.187 |
| **Maternal characteristics at time of vaccination** | | | | | | |
| Age | 568 | 23.0 (4.8) | 0.7298 | 535 | 23.2 (4.6) | 0.8115 |
| Gestational age at vaccination | 568 | 24.0 (5.1) | 0.0018 | 535 | 23.6 (5.2) | 0.1443 |
| 17-<25 |  | 59.5 | <0.001 |  | 63.6 | 0.544 |
| 26-<35 |  | 40.5 |  |  | 36.5 |  |
| Systolic BP, at enrollment | 567 | 100.3 (9.9) | 0.7669 | 534 | 101.0 (9.8) | 0.1301 |
| Diastolic BP, at enrollment | 567 | 67.1 (9.1) | 0.1003 | 534 | 67.4 (8.7) | 0.05 |
| Height, at enrollment | 568 | 151.5 (5.8) | 0.4092 | 533 | 151.2 (5.3) | 0.0146 |
| Weight, at enrollment | 568 | 48.2 (6.9) | 0.6845 | 533 | 48.5 (7.7) | 0.5303 |
| BMI, at enrollment | 568 | 21.0 (2.8) | 0.2691 | 532 | 21.2 (2.9) | 0.5597 |
| <18.5 |  | 17.4 | 0.596 |  | 14.3 | 0.428 |
| 18.5-<25 |  | 75.2 |  |  | 76.5 |  |
| 25-<30 |  | 6.7 |  |  | 7.7 |  |
| 30+ |  | 0.7 |  |  | 1.5 |  |
| No education | 531 | 44.4 | 0.115 | 492 | 45.9 | 0.074 |
| Nulliparous | 597 | 46.7 | 0.025 | 566 | 44.0 | 0.128 |

Included are those missing height & weight data from infeasibility or measurement taken <150 days or >=210 days or just missing, all live births.

*Statistical difference in characteristic with those who had anthropometry data between ages 150-<210 days (comparing placebo with data vs. placebo without data, comparing vaccinated with data vs. vaccinated without data).

Supplemental Table 2: Vaccine effect with imputed 6-month weights and lengths

|  | Mean or % (SD) | | Beta / RR (95% CI) |
| --- | --- | --- | --- |
|  | placebo (N=1596) | vaccine (N=1601) |  |
| Weight (g) | 6821 (29) | 6844 (28) | 22.6 (-53.2, 98.4) |
| Weight for length |  |  |  |
| <-2 Z-scores | 19.5 (1.1) | 19.6 (1.1) | 1.01 (0.87, 1.17) |
| <-3 Z- scores | 5.6 (0.6) | 4.5 (0.6) | 0.81 (0.59, 1.11) |
| Length for age |  |  |  |
| Length (cm) | 64.5 (0.07) | 64.6 (0.07) | 0.09 (-0.10, 0.28) |
| <-2 Z-scores | 16.2 (1.1) | 15.2 (1.0) | 0.94 (0.79, 1.12) |
| <-3 Z- scores | 3.5 (0.5) | 2.4 (0.4) | 0.68 (0.44, 1.04) |
